# Proteus empyema as a rare complication from an infected renal cyst, a case report

**DOI:** 10.1186/s12890-020-01346-w

**Published:** 2020-11-27

**Authors:** Kranthikiran Earasi, Caitlin Welch, Adam Zelickson, Clinton Westover, Chintan Ramani, Cameron Sumner, Eric M. Davis

**Affiliations:** 1 Department of Medicine, University of Virginia, 1714 Calvary Circle, Apt. 302, Charlottesville, VA 22911 USA; 2 Division of Pulmonary and Critical Care Medicine, Department of Medicine, University of Virginia, Charlottesville, VA USA; 3 Department of Radiology, University of Virginia, Charlottesville, VA USA; 4 Department of Pathology, University of Virginia, Charlottesville, VA USA; 5 Department of Anesthesiology, University of Virginia, Charlottesville, VA USA

**Keywords:** Empyema, Proteus, Renal cyst, Case report, Computed tomography

## Abstract

**Background:**

The most commonly isolated organisms in a parapneumonic effusion include *S. pneumoniae, H. influenzae*, and *S. aureus*. If unusual organisms are isolated from the pleural space, further investigation is warranted to locate the primary source. We present a patient with an infected chronic renal cyst found to have an empyema secondary to *Proteus mirabilis* to highlight the importance of further diagnostic workup when encountering unusual organisms in the pleural space.

**Case presentation:**

A 40-year-old African-American female, with a past medical history of asthma and sickle cell trait, presented with 5 weeks of upper respiratory tract symptoms and chest pain. A computed tomography angiogram (CTA) of the chest was negative for a pulmonary embolism but revealed a loculated left sided pleural effusion with associated left-lower lobe consolidation. She was started on empiric antibiotics, and a chest tube was inserted with drainage of frank pus. Fluid gram stain was positive for gram negative rods.

Intrapleural fibrinolytics were administered for 72 h given the presence of loculations. With no improvement following fibrinolytics, she was taken to the operating room for large bore chest tube placement and left visceral pleura decortication. Pleural fluid cultures speciated to *Proteus mirabilis*, so further cross-sectional imaging of her abdomen/pelvis was pursued to evaluate for a primary source. A complex cystic lesion in the upper pole of the left kidney that communicated with the ipsilateral diaphragm was identified. Subsequent drainage and culture of the renal cyst was positive for *Proteus mirabilis*. Given clinical improvement following these interventions she was discharged with an extended course of antibiotics with plans for repeat imaging following completion of treatment.

**Conclusions:**

While cases of *Proteus mirabilis* empyema have previously been reported as a consequence of conditions such as pyelonephritis, we present, to our knowledge, the first case of a *Proteus mirabilis* empyema as a consequence of an infected renal cyst communicating with the pleural space. This study highlights that further evaluation with cross-sectional imaging is warranted when unusual organisms are found in the pleural space. Anatomic abnormalities that become apparent on imaging may help elucidate the source of infection.

## Background

The most commonly isolated organisms in a parapneumonic effusion include *S. pneumoniae, H. influenzae*, and *S. aureus* [1]. If unusual organisms are isolated from the pleural space, further investigation is warranted to locate the primary source. While *Enterobacteria*, *Pseudomonas spp*., and *M. tuberculosis* may comprise some of these unusual organisms, some of which are more commonly found in nosocomial infections, the gram negative bacteria *Proteus mirabilis* may also be isolated [2–4]. Known as one of the leading causes of pyelonephritis and urolithiasis, few case studies exist reporting its presence in the pleural space, and those present only describe the presence of this bacteria in the pleural space in the setting of an underlying pyonephrosis/pyelonephritis [5–7]. To our knowledge, there has been no association between *Proteus* related pleural disease and chronic renal disease. We present a patient with an infected chronic renal cyst found to have an empyema secondary to *Proteus mirabilis* to highlight the importance of further diagnostic workup when encountering unusual organisms in the pleural space.

## Case presentation

A 40-year-old African American female presented to our institution with 5 weeks of upper respiratory tract symptoms and chest pain. Her past medical history was significant for asthma, type 2 diabetes, hypertension, and sickle cell trait. Prior to admission, she was seen at urgent care centers and her symptoms were attributed to a viral illness and supportive care was recommended. Given worsening dyspnea on exertion, she presented to the emergency room for further evaluation. Initial vital signs were notable for a temperature of 100.5**°** Fahrenheit, heart rate of 107, respiratory rate of 30 breaths per minute, blood pressure of 126/75, and an oxygen saturation of 95% on 2 l per minute of supplemental oxygen. Physical examination on arrival was notable for diaphoresis, tachypnea, diminished breath sounds in the left lung base as well as dullness to percussion over the left lower lung field, and tenderness to palpation in the left upper quadrant of the abdomen. Workup with a computed tomography angiogram (CTA) of the chest did not show evidence of a pulmonary embolism but was notable for a loculated left sided pleural effusion with associated left lower lobe consolidation. She was started on ceftriaxone and azithromycin empirically, and a chest tube was inserted which resulted in drainage of frank pus. Fluid analysis showed a WBC of 210,200 cells/uL, LDH of 12,915 units/L, and a pleural fluid pH of 6.2. A gram stain was positive for gram negative rods.

**Fig. 1:**
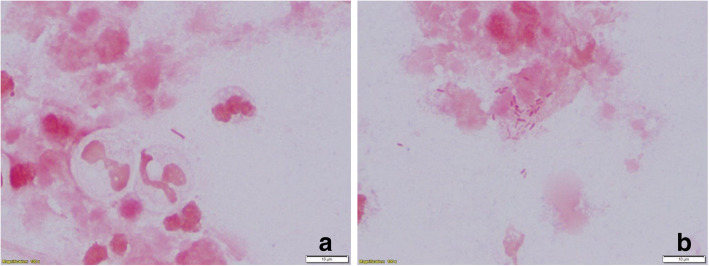
Microscopic view of *Proteus mirablils* in the patient’s pleural fluid with the black arrows indicating *Proteus* species. (**a and b**)

**Fig. 2:**
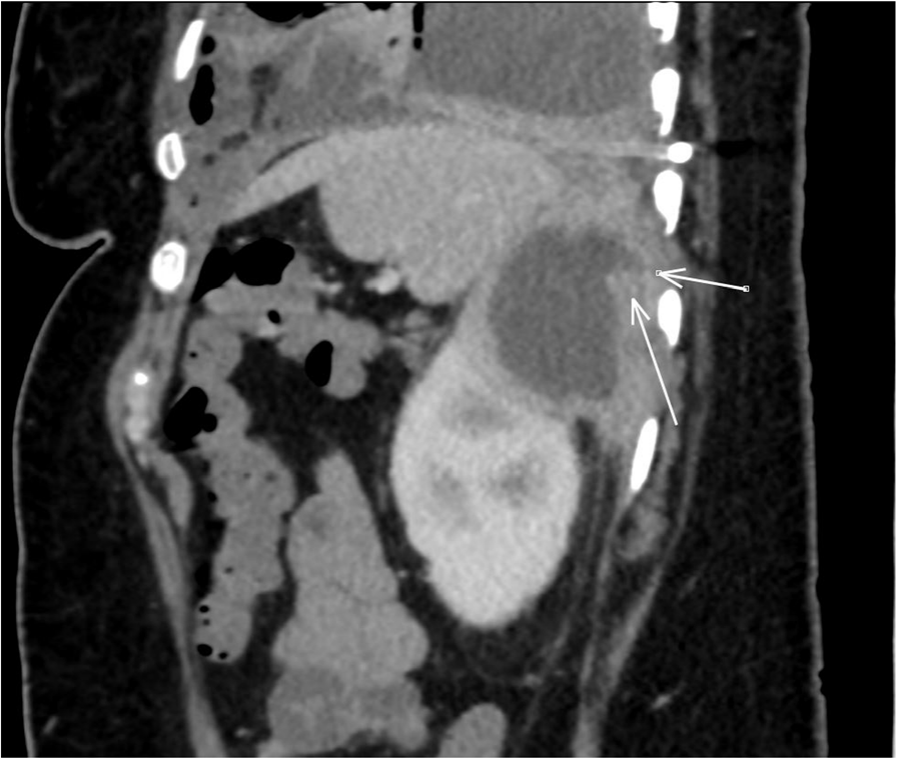
Computed Tomography results showing a sagittal cross section of the patient’s abdomen with the white arrows indicating the renal cyst and tract extending posteriorly to the diaphragm

**Fig. 3:**
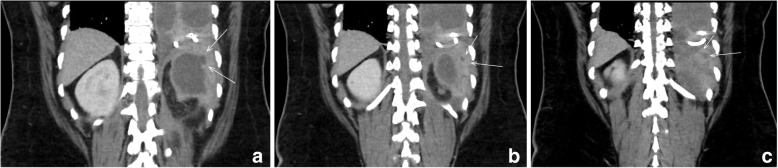
Computed Tomography results showing sequential coronal cross sections of the patient’s abdomen extending posteriorly with the white arrows indicating the posteriorly and superiorly extending tract from the renal cyst to the diaphragm. (Anterior to Posterior (Left to Right): **a, b, and c**)

Intrapleural fibrinolytics with tissue plasminogen activator (tPA) and deoxyribonuclease (DNAse) were administered for 72 h given the presence of loculations. With no significant improvement following fibrinolytics, she was taken to the operating room for large bore chest tube placement and video-assisted thoracoscopic surgery (VATS) decortication of the left visceral pleura. Pleural fluid cultures speciated to *Proteus mirabilis* (Fig. 1), so she underwent further imaging to evaluate for a primary source. Computed tomography (CT) abdomen/pelvis revealed a complex cystic lesion in the upper pole of the left kidney that communicated with the ipsilateral diaphragm (Figs. 2 and 3). Urinalysis on presentation was unremarkable. A drainage catheter was placed within this abscess, and culture of the cyst fluid was also positive for *Proteus mirabilis*.

The patient clinically improved following these interventions and was transitioned to intravenous ceftriaxone and metronidazole following culture speciation. At the completion of a 12 day hospitalization, she was discharged to home on an extended course of oral amoxicillin-clavulanate. Repeat CT chest and CT abdomen/pelvis 18 days post discharge, following the completion of the antibiotic course, showed interval resolution of the renal cyst and left sided empyema.

## Discussion and conclusions

While cases of *Proteus mirabilis* empyema have previously been reported as a consequence of conditions such as pyelonephritis, we present, to our knowledge, the first case of a *Proteus mirabilis* empyema as a consequence of an infected renal cyst communicating with the pleural space. A prior case series identified *Proteus mirabilis* in the pleural fluid of three separate patients who had effusions secondary to either metastatic malignancy or heart failure. Despite the different etiologies, all three effusions were alkalotic with an average pH of 7.77 [8]. The alkalinity of the fluid was hypothesized to be the result of the urease producing ability of *Proteus*. The measurement of pleural fluid pH along with pleural ammonia levels were thought to be of diagnostic utility when considering *Proteus* as a causative organism [8]. Though our patient’s pleural fluid pH was 6.2, the use of procedural lidocaine may explain this discrepancy from the findings of the aforementioned study [9].

Our case presented an otherwise healthy female with few comorbidities who was found to have an empyema secondary to *Proteus*. Despite the infected chronic renal cyst, her lack of urinary symptoms or abnormal urinalysis is consistent with prior cases of *Proteus*-related lung infections, demonstrating the importance of considering an intra-abdominal source of infection in these cases [10].

This study highlights that further evaluation with cross-sectional imaging should be considered when unusual organisms are found in the pleural space. Anatomic abnormalities that become apparent on imaging may help elucidate the source of infection. In combination with laboratory markers, radiologic findings can prove to be of equal importance in guiding treatment.

## Data Availability

No raw data was utilized in the production of this manuscript. Only information pertaining to this patient’s hospital course as documented in our institution’s electronic medical record was utilized in the creation of this work.

## Abbreviations

CTA: Computed tomography angiogram
CT: Computed tomography
DNAse: Deoxyribonuclease
LDH: Lactate Dehydrogenase
tPA: Tissue plasminogen activator
VATS: Video-assisted thoracoscopic surgery
WBC: White Blood Cell
